# Application of the novel estimation method by shear wave elastography using vibrator to human skeletal muscle

**DOI:** 10.1038/s41598-020-79215-z

**Published:** 2020-12-17

**Authors:** Wakako Tsuchida, Yoshiki Yamakoshi, Shingo Matsuo, Mayu Asakawa, Keita Sugahara, Taizan Fukaya, Eiji Yamanaka, Yuji Asai, Naotaka Nitta, Toshihiko Ooie, Shigeyuki Suzuki

**Affiliations:** 1 Department of Life Science and Biotechnology, Health and Medical Research Institute, National Institute of Advanced Industrial Science and Technology (AIST), 2217-14 Hayashi-cho, Takamatsu, Kagawa 761-0395 Japan; 2 Graduate School of Science and Technology, Gunma University, 1-5-1, Tenjin-cho, Kiryu, Gunma 376-8515 Japan; 3 Department of Rehabilitation, Faculty of Health Sciences, Nihon Fukushi University, 26-2 Higashihaemi-cho, Handa, Aichi 475-0012 Japan; 4 Department of Rehabilitation, Matsunami General Hospital, 185-1 Dendai, Kasamatsu-cho, Hashima , Gifu 501-6062 Japan; 5 Medical Corporation Sanaikai, 2-2-1 Tada, Obama, Fukui 917-0026 Japan; 6 Institute for Human Movement and Medical Sciences, Niigata University of Health and Welfare, 1398 Shimami-cho, Kita-ku, Niigata, 950-3198 Japan; 7 Department of Rehabilitation, Kyoto Kujo Hospital, 10 Karahashirajoumon-cho, Minami-ku, Kyoto, 601-8453 Japan; 8 Department of Rehabilitation Medicine, Tokyo Bay Rehabilitation Hospital, 4-4-1 Yatsu, Narashino, Chiba Japan; 9 Department of Health and Sports Sciences, School of Health Sciences, Asahi University, 1851 Hozumi, Mizuho, Gifu 501-0296 Japan

**Keywords:** Physical examination, Skeletal muscle

## Abstract

In recent years, non-invasive measurement of tissue stiffness (hardness) using ultrasound elastography has attracted considerable attention. It has been used to evaluate muscle stiffness in the fields of rehabilitation, sports, and orthopedics. However, ultrasonic diagnostic devices with elastography systems are expensive and clinical use of such devices has been limited. In this study, we proposed a novel estimation method for vibration-based shear wave elastography measurement of human skeletal muscle, then determined its reproducibility and reliability. The coefficient of variation and correlation coefficient were used to determine reproducibility and reliability of the method by measuring the shear wave velocities in konjac phantom gels and agar phantom gels, as well as skeletal muscle. The intra-day, day-to-day, and inter-operator reliabilities were good when measuring the shear wave velocities in phantom gels. The intra-day and day-to-day reliabilities were good when measuring the shear wave velocities in skeletal muscle. The findings confirmed adequate reproducibility and reliability of the novel estimation method for vibration-based shear wave elastography. Therefore, the proposed measurement method may be a useful tool for evaluation of muscle stiffness.

## Introduction

Objective measurement of muscle tissue elasticity can help to confirm the pathological states of various diseases associated with skeletal muscle stiffness and aid in determining treatment efficacy. In clinical practice, muscle spasticity after stroke, spinal cord injury, multiple sclerosis, and myopathy are usually assessed subjectively through manual palpation to characterize muscle tissue. Other characteristics assessed in this manner include tissue stiffness in myofascial pain syndrome, neck–shoulder pain, and low back pain, as well as rehabilitation progress. Muscle characteristics can be measured using ramp-and-hold tests, pendulum tests, and dynamometry; these approaches provide valuable information concerning surrounding tissues but cannot isolate the mechanical properties of individual muscles from those of associated skin, subcutaneous tissues, tendons, neurovascular structures, and joint capsules^1–3^. Although distinct terms are used in different fields of clinical practice, the muscle characteristics described by these terms are related to muscle stiffness. One of the most important parameters used to quantify soft tissue stiffness (or elasticity) is the modulus of elasticity, also known as Young’s modulus; this parameter is defined as the slope of the stress–strain curve of a material in the elastic deformation region, which comprises a local mechanical property of the constituent material. Hence, quantification of elastic modulus distribution could improve the accuracy of muscle stiffness measurement.

A method known as ultrasound strain elastography has been used to assess skeletal muscle stiffness. In this method, an ultrasonic diagnostic probe is held to the body surface and minimal repetitive pressure is applied to tissues. This pressure generates real-time strain images using a transducer. Accordingly, when a consistent amount of pressure is applied, softer tissues demonstrate a greater amount of deformation and therefore experience more strain, compared with stiffer tissues. However, tissue stiffness is measured as a ratio of the amount of actual strain to target strain, indicating that quantitative measurement using Young’s modulus remains impossible, although semiquantitative measurement is possible. At least three cycles of compression and decompression are recommended for optimal measurement^4^; however, extensive tissue preload through repeated compressions can alter tissue elasticity. Notably, this measurement procedure requires specific technical skills, such that the measurement accuracy depends on the examiner’s experience^5–8^.

Recently, an approach that utilizes shear wave elastography has attracted attention as a new method for quantitative estimation of tissue stiffness. Shear wave propagation speed is highly dependent on tissue stiffness; Doppler ultrasonography can be used to measure tissue stiffness because it can measure small propagation-related displacement fluctuations with relatively high accuracy^9,10^. In ultrasound shear wave elastography, shear waves can be launched in a controlled manner by external mechanical vibrations^11–22^ and acoustic radiation force impulses^23–28^. The use of an external vibrating source is much less common among commercial ultrasound shear wave elastography systems because it is relatively difficult to image the target tissue at appropriate depth; moreover, the required devices can be cumbersome^11,22^. Most commercial ultrasound shear wave elastography systems (e.g., Philips, GE Healthcare, Siemens, Canon, Mindray, Samsung, and Supersonic) use acoustic radiation force impulse-based techniques to generate ultrasonic shear waves in tissues^27,28^. This method requires strong ultrasonic waves, but undesirable temperature elevation can occur if bone and surrounding tissues are located near the focal point^29^. One major limitation of this method is that the detection of shear waves generally requires ultrasound imaging equipment with a high frame rate; these systems are expensive. Therefore, it is difficult to introduce such systems in general clinical practice, rehabilitation facilities, and sports medicine clinics.

Accordingly, external mechanical vibration has been proposed as a cost-effective alternative method for shear-wave induction, with the goal of enabling wider adoption of shear wave elastography^14,17,20,22^. Recently, there has been considerable progress in developing the technological foundation required for external mechanical vibration-based shear wave elastography imaging^16,19,30^. Vibro-elastography has found broad clinical applications in prostate cancer detection and liver fibrosis staging^20,31,32^. To the best of our knowledge, there is no widely available method that can visualize the shear wave wavefront in real time by means of conventional external vibration-based measurement. A shear wave that is reflected from the tissue boundary produces a standing wave in the vicinity of the boundary, which reduces the accuracy of shear wave velocity estimation. The complicated propagation of the shear wave inside tissue also diminishes measurement accuracy. Therefore, we presume that confirmation of the propagation wavefront (i.e., propagation direction, reflection, refraction, and complicated wavefront) of the shear wave and measurement of the shear wave velocity will facilitate quantitative measurements with good reproducibility.

Color Doppler shear wave imaging is a novel wave front imaging method in which ultrasound color flow images are used to detect the wave fronts of shear waves; this approach allows direct, real-time observation of the wave fronts of shear waves^33–35^. Shear wave elastography uses continuous shear waves generated by a mechanical vibrator attached to the tissue surface. The addition of a very simple device system to a traditional ultrasound device allows quantitative visualization of tissue stiffness; this offers an easy-to-use method for evaluation of the viscoelastic properties of superficial tissues. However, the adaptation of this system to skeletal muscle has not yet been investigated in detail. Therefore, this study was performed to examine the reproducibility and reliability of a novel estimation method for vibration-based shear wave elastography measurement of human skeletal muscle.

## Results

### Evaluation of reproducibility when applying the novel estimation method to vibration-based shear wave elastography measurement of phantom gels

**Figure 1:**
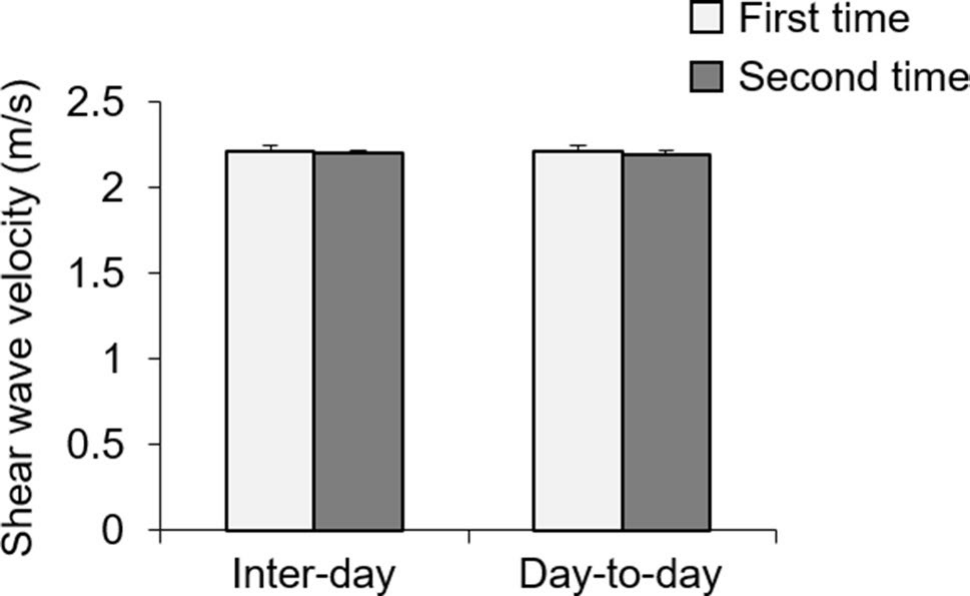
Shear wave velocities in konjac yam phantom jelly. Konjac yam phantom jelly was measured twice per day for 2 days with a between-measurement interval of 1 day. Data are presented as mean ± standard deviation (five measurements per trial).

The mean shear wave velocities in konjac yam phantom jelly were 2.21 ± 0.03 m/s (first measurement: coefficient of variation [CV], 1.38%) and 2.21 ± 0.01 m/s (second measurement: CV, 0.38%) on the first day, and 2.21 ± 0.02 m/s (first measurement: CV, 0.83%) and 2.28 ± 0.02 m/s (second measurement: CV, 0.78%) on the second day (Fig. 1). The intra-day, day-to-day, and inter-operator CV values of konjac yam phantom jelly elasticity were 0.2%–0.6%, 0.6%–0.7%, and 0.5%–0.7%, respectively.

**Figure 2:**
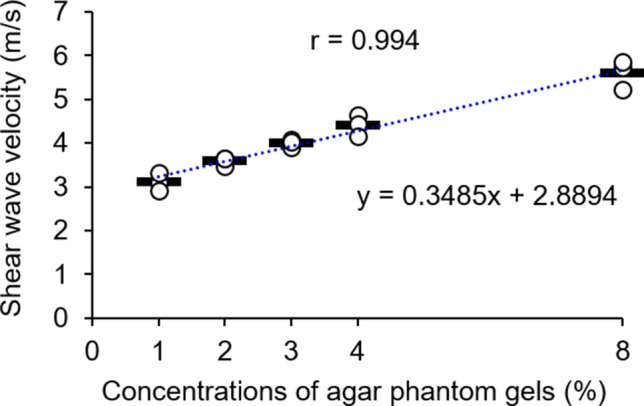
Shear wave velocities in agar phantom gels. The shear wave velocities in agar phantom gels (1%, 2%, 3%, 4%, and 8%) were measured three times. Horizontal bars indicate mean values.

In analyses using the agar gel, the shear wave velocity increased as the agar concentration increased from 1 to 8%. Moreover, the agar concentration and shear wave velocity were positively correlated (r = 0.99; Fig. 2). Table 1 shows the CV and intra-class correlation coefficient (ICC) values of the intra-day (CV, < 4.8; ICC, 0.98), day-to-day (CV, < 7.7; ICC, 0.96), and inter-operator reliabilities of agar phantom gel elasticity (CV, < 4.0; ICC, 0.98).

**Table 1:** Intra- and inter-operator reliabilities of agar phantom gel elasticity.

	Intra-day reliability	Day-to-day reliability	Inter-operator reliability
ICC (95% CI)	0.98 (0.80–1.00)	0.96 (0.65–1.00)	0.98 (0.65–1.00)
CV	1.8–4.8	0.6–7.7	1.6–4.0

The intra-class correlation coefficient (ICC), 95% confidence interval (CI), and coefficient of variation (CV) were calculated for each trial. Phantom gels with different concentrations of agar (1%, 2%, 3%, 4%, and 8%) were measured twice per day for 2 days with a between-measurement interval of 1 day (three measurements per trial).

### Evaluation of reproducibility when applying the novel estimation method to vibration-based shear wave elastography measurement of skeletal muscle

**Figure 3:**
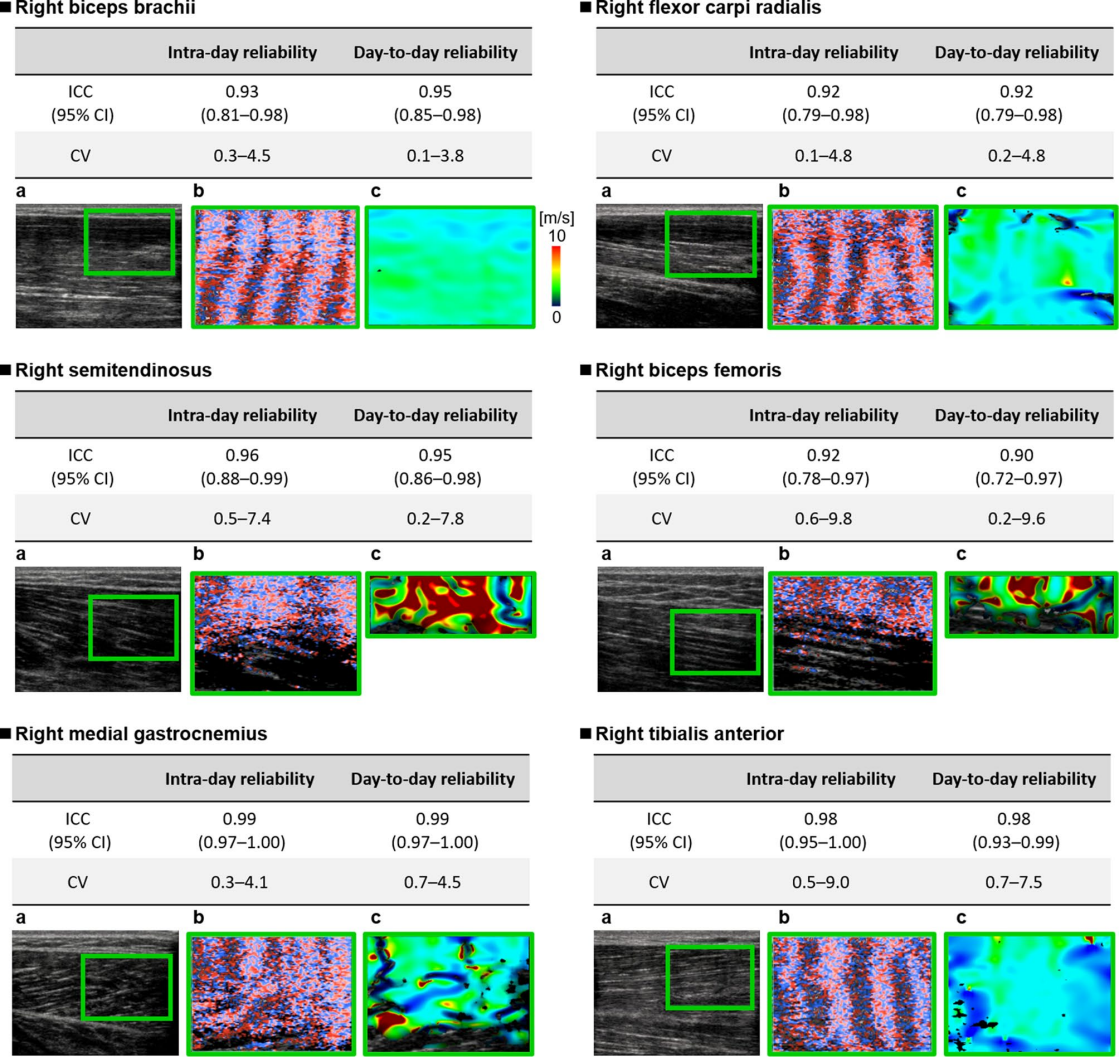
Intra-day and day-to-day reliabilities of shear wave velocity estimation of skeletal muscle stiffness. **(a)** B-mode image, (b) color flow image, **(c)** shear wave velocity map. The right biceps brachii, flexor carpi radialis, semitendinosus, biceps femoris, medial gastrocnemius, and tibialis anterior were measured twice per day for 2 days with a between-measurement interval of 1 day (three measurements per trial). For the semitendinosus and biceps femoris, statistical analysis focused on the upper half of the selected area. Results of skeletal muscle shear wave velocity (mean ± standard deviation) are shown in Supplementary Fig. S1 online.

Figure 3 shows the CV and ICC values of the intra-day (CV, < 9.8; ICC, > 0.92) and day-to-day (CV, < 9.6; ICC, > 0.90) reliabilities of shear wave velocity estimation of skeletal muscle stiffness. It also shows example color flow images and shear wave velocity maps for the biceps brachii, flexor carpi radialis, semitendinosus, biceps femoris, medial gastrocnemius, and tibialis anterior.

## Discussion

Our study provides evidence of excellent reliability when using our novel estimation method for vibration-based shear wave elastography measurements of phantom elasticity and skeletal muscle stiffness. These observations are clinically important because skeletal muscle stiffness is observed in various conditions, such as myofascial pain syndrome^36^, neck and shoulder pain^27^, and low back pain^37^. Thus, our results suggest that multiple patient groups who experience muscle stiffness may benefit from our proposed method, which is a useful tool for evaluating diseases characterized by muscle stiffness and determining treatment effects; this method is expected to help reduce costs and promote introduction of vibration-based shear wave elastography in general clinical practice.

The proposed estimation method for vibration-based shear wave elastography demonstrated excellent intra-day, day-to-day, and inter-operator reliabilities when measuring phantom elasticity. Konjac gel is used as a phantom for ultrasound imaging^38,39^ and elastography^34^. The mean shear wave velocity in konjac phantom gel was 2.2 m/s, which is reasonably consistent with the findings in a previous study^34^. The CV calculated from the mean and standard deviation of five measurements during one trial was 0.38%–1.38%, indicating excellent repeatability for measurements of phantom elasticity. Therefore, triplicate measurements during each trial were performed in subsequent experiments.

Intra-day and day-to-day reliabilities were measured to evaluate temporal variation. These results revealed that our proposed method was able to reliably measure elasticity at two different time points (intra-day CV, 0.2%–0.6%; day-to-day CV, 0.6%–0.7%). Furthermore, inter-operator reliability was measured to evaluate operator variation; the findings showed that two independent operators produced reliable elasticity measurements using our novel method (inter-operator CV, 0.5%–0.7%).

To verify the effectiveness of our proposed method, agar gels were used because agar is economical and because elasticity can be easily manipulated by modifying the agar concentration^34,40^. Several attempts were made to produce different concentrations of agar phantom gels, which were prepared based on the recipes described in Table 2. These recipes ensured that the respective components did not separate, powdered agar was dissolved in the solution, and the agar was uniformly solidified. Our results revealed that, as the concentration of agar increased, shear wave velocity increased; moreover, the agar concentration was positively correlated with shear wave velocity. In addition, the CV and ICC measurements were consistent among different agar concentrations, revealing good intra-day, day-to-day, and inter-operator reliabilities. Therefore, our novel estimation method for shear wave elastography may be reliable for measurement of elasticity.

**Table 2:** Recipes for agar phantom gel preparation.

Concentration (%)	Powdered agar (g)	Sterilized milk (ml)
1	4	396
2	8	392
3	12	388
4	16	384
8	32	368

Phantom gels with different concentrations of agar (1%, 2%, 3%, 4%, and 8%) were mixed with sterilized milk.

The most striking finding of this study was that our estimation method for shear wave elastography had excellent intra-day and day-to-day reliabilities when measuring the shear wave velocities in the right biceps brachii, flexor carpi radialis, semitendinosus, biceps femoris, medial gastrocnemius, and tibialis anterior. This finding suggests that our method can reliably assess muscle elasticity.

Other authors have proposed alternative techniques to measure muscle stiffness in healthy skeletal muscle by means of ultrasound elastography^27,41–45^. It is difficult to compare shear wave velocities measured in our study with values measured in previous studies because of inconsistencies in probe positioning, variations in ultrasound elastography techniques, and differences in the methods used to report stiffness. Available shear wave elastography measurement parameters include shear wave speed, elastic modulus, shear elastic modulus, Young’s modulus, and elasticity^44–47^. Notably, Ballyns et al.^41^ measured biceps brachii shear wave velocity by means of external vibrations. Although their probe and vibrator positions, frequencies, and analytical methods differed from our method, the overall approaches were similar in their study and our study; thus, we presumed that their results could be readily compared with our findings. In the study by Ballyns et al., measurements were performed at 60, 110, 160, and 200 Hz; measurements at 276 Hz (the frequency used in our study) were not directly reported. However, the data provided in their publication implied that biceps brachii shear wave velocity at 276 Hz would be approximately 4 m/s, similar to our measured values of 3.02–5.12 m/s (see Supplementary Fig. S1 online). Therefore, the shear wave velocity measured by the proposed method is similar to the value reported in a previous study that involved external vibration measurements.

Our findings are comparable with those of previous studies that have shown good/excellent reliability in measuring muscle elasticity by means of shear wave elastography in resting muscles and during active contraction^48–50^. Lacourpaille et al.^48^ investigated the reliability of elastic modulus in resting muscles by means of shear wave elastography using supersonic shear wave imaging. They reported good intra-session (n = 20) and inter-day (n = 21) reliabilities (biceps brachii ICC, 0.868 and 0.832, respectively; medial gastrocnemius ICC, 0.950 and 0.922, respectively; and tibialis anterior ICC, 0.811 and 0.809, respectively). Depending on the muscle, the mean temporal variability in the CV over 10 measurements ranged from 2.9% to 5.0%. Dubois et al.^49^ examined the intra-operator repeatabilities of a measurement protocol to assess shear modulus in the resting lower limb muscles of 10 healthy participants who underwent shear wave elastography using supersonic shear wave imaging. They reported poor repeatability in the biceps femoris (CV, 18%), semimembranosus (CV, 15%), semitendinosus (CV, 20%), and medial gastrocnemius (CV, 9%) muscles. In that study, the CV of shear modulus was calculated and measured six times. Koo et al.^50^ performed shear wave elastography using supersonic shear wave imaging to estimate muscle elasticity in the resting tibialis anterior of 20 healthy participants, then calculated the CV of shear elastic modulus in triplicate. They observed excellent test–retest reliability (ICC , 0.942).

In this study, we confirmed adequate reproducibility and reliability using our novel estimation method for vibration-based shear wave elastography measurement of skeletal muscle. To verify inter-day and day-to-day reliabilities, we measured participants’ major surface muscles (i.e., biceps brachii, flexor carpi radialis, semitendinosus, biceps femoris, medial gastrocnemius, and tibialis anterior) twice per day for 2 days, with a between-measurement interval of 1 day. For all muscles, the intra-day (CV, < 9.8; ICC, > 0.92) and day-to-day (CV, < 9.6; ICC, > 0.90) reliabilities of the novel estimation method for skeletal muscle were good. Compared with the results of prior studies^48–50^, our findings indicate that the proposed method is a highly reliable approach for estimating major muscle elasticity.

Good reliability in our study may have been achieved through standardization of the scanning site with reference to body landmarks, imaging parameters, and both location and size of the region of interest. In addition, the positions of the probe and excitation point were adjusted to ensure that shear waves propagated in the direction of muscle fibers. Muscle stiffness at rest is perhaps the simplest measurement to capture using ultrasound elastography, but this method must be performed in a standardized manner. In particular, the orientation of the transducer probe affects measurement reliability^46^. Because our method can directly show shear wave wavefronts in real time, the observer can easily identify the optimal probe orientation and/or excitation point, then reproduce shear wave wavefronts that propagate uniformly in the region of interest; these approaches facilitate good reliability when measuring muscle stiffness.

### Limitations

There were several notable limitations in the present study. First, we did not investigate correlations with other quantitative methods in this study, because we previously determined the effectiveness of the new estimation method by comparison with values measured using other instruments. Moreover, we previously showed that shear wave velocity measurements in phantom gels, supraclavicular muscles, and trapezius muscles acquired using the new method were consistent with those acquired by means of other instruments^15,33,34,51,52^. Comparison of shear wave velocity among skeletal muscle sites and quantitative methods is an important consideration for clinical application of novel skeletal muscle stiffness measurement methods and devices.

Second, all participants were asymptomatic young male volunteers and elasticity was measured in static muscles. Thus, the findings may not be generalizable to a variety of patients or muscles. Nevertheless, the findings provide important support for further validation of this technique’s reliability under different conditions. In particular, this technique should be performed in asymptomatic patients and the findings should be compared with measurements in patients who exhibit various symptoms or pathologies.

Third, only the elasticity of the middle portion of the muscle was examined, on the basis of the recommended placement of surface electrodes for standardized electromyography measurement. Although we focused on sites that are often measured in clinical practice, our results were highly reproducible and reliable in all parts of skeletal muscle measured in this study. Moreover, the results of this study provide important guidance for clinical application of our method. In the future, we plan to investigate areas other than the central section of the muscle, as well as muscle–tendon transition zones, to determine differences in shear elastic modulus among these sites.

Finally, only the elasticity of major surface muscles was examined, largely because these muscles are frequently measured in clinical practice. As noted above, our results were highly reproducible and reliable in the measured muscles. In the future, we plan to investigate other muscles (i.e., deep tissues) to support clinical implementation of our method. As shown in the photograph of the hamstring (Fig. 3), tissues can be readily measured close to the body surface; however, the measurement of deep tissues may be difficult. A previous study^51^ showed that stable measurement of deeper tissues may be achieved by modifying the shear wave frequency of the actuator with respect to shear wave tissue absorption. However, the feasibility of this approach must be confirmed through additional investigations.

## Conclusions

In this experimental study, we assessed phantom gels and volunteers’ skeletal muscles by means of vibration-based shear wave elastography. Our findings confirmed adequate reproducibility and reliability of a novel estimation method for shear wave elastography measurement of skeletal muscle at sites commonly assessed in clinical practice. Depending on the results of additional validation studies in study populations with different ethnicities and disease states, the proposed measurement method may constitute a useful tool for evaluation of muscle stiffness in both clinical and research settings.

## Methods

### Experimental system

**Figure 4:**
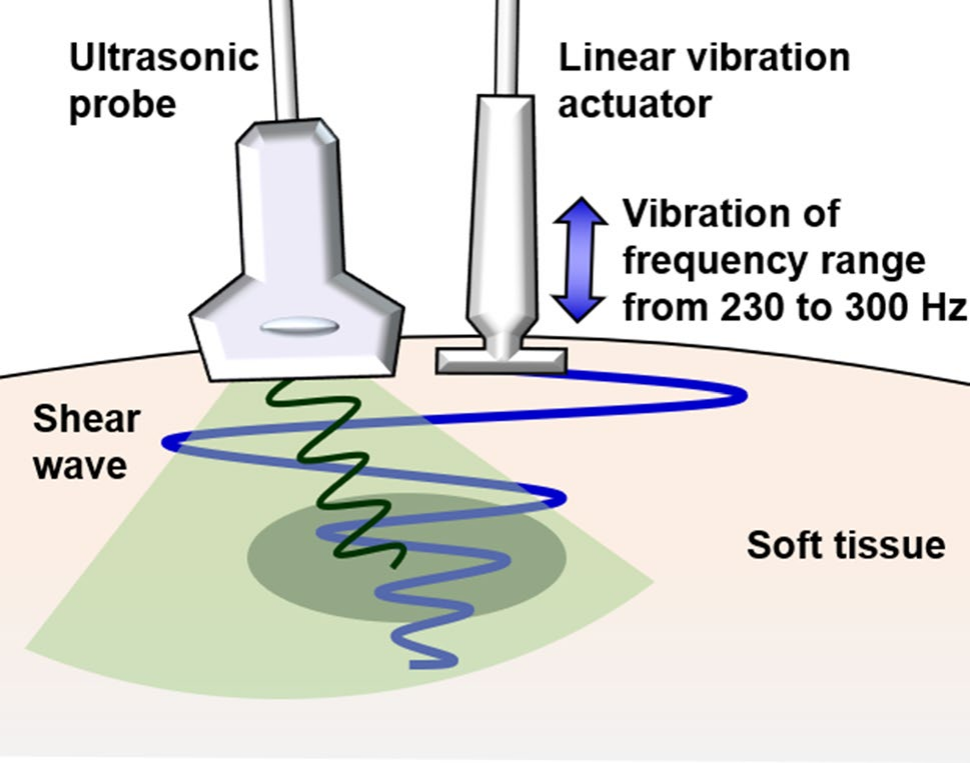
Schematic of color Doppler shear wave imaging.

Figure 4 illustrates the experimental system. Our novel estimation method for shear wave elastography was implemented using ultrasound scanners (Xario SSA-660A, Toshiba, Japan). The shear wave imaging method was previously described; quantitative evaluation confirmed a good correlation between the small displacement estimation method and shear wave velocity measured using our new method^15,33,34^. Notably, shear wave velocity is derived from stiffness by assuming that shear viscosity is negligible. Thus, comparative experiments between these two methods for human supraspinatus muscle, trapezius muscle, and an agar gel phantom have been performed; shear wave velocities measured using our novel estimation method were consistent with measurements performed by means of acoustic radiation force impulses^34,51,52^. In our novel estimation method, the shear wave frequency is selected from among several frequencies that satisfy the shear wave frequency conditions. The spatial resolution of a shear wave map decreases upon selection of a low frequency. However, determination of shear wave displacement amplitude is difficult because of the limited vibrator output power. Hence, shear wave frequency in this study was set within the frequency range from 235.8 to 296.6 Hz, depending on the pulse repetition frequency of the ultrasound scanner to satisfy the shear wave frequency condition of m = 1.

**Figure 5:**
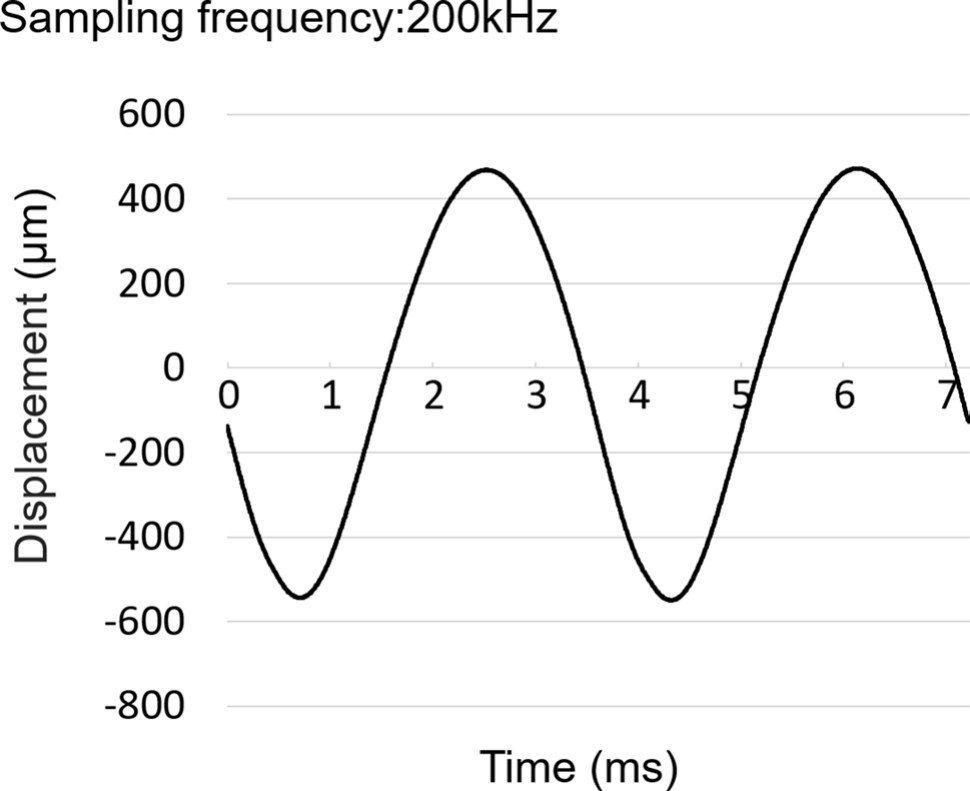
Acceleration data demonstrating linear vibration motor displacement when positioned over skeletal muscle.

A short (45 mm) and lightweight (30 g) linear vibration motor, designed to manipulate an electric toothbrush, was adopted for the actuators by generating vibrations with a displacement amplitude of > 400 μm and frequency range of 230–300 Hz. Displacement of the linear vibration motor was measured using a microelectromechanical system accelerometer (ADXL001, Analog Devices, USA) at an accelerometer sampling frequency of 200 kHz; the displacement was derived by two-step integration. Figure 5 shows the vibration displacement when positioned over skeletal muscle. The harmonic distortion was measured by Fourier analysis. Power spectrum analysis showed that the second and third harmonic components of displacement were − 44.5 dB and − 79.9 dB at a vibration frequency of 276 Hz and a displacement amplitude of 300 μm. This result was considered negligible and the harmonic distortion was therefore ignored. Hence, this actuator was presumed to generate a sinusoidal waveform that was suitable for the novel estimation method.

To reconstruct shear wave maps, the color flow image on the ultrasound scanner was recorded as a moving picture for 2 s. Then, the color flow image was fed into a computer by means of a video capture device. Fourier analysis and a directional filter were applied; shear wave phase, velocity, and propagation maps were reconstructed. The central processing unit time required for image reconstruction was < 1 s. In our experimental setup, shear wave maps were reconstructed at intervals of 4 s until the stop button on the computer screen was clicked.

### Participants

Fourteen healthy male college students voluntarily participated in the study. Written informed consent was obtained prior to the first measurement and all study procedures related to human participants were approved by the Human Research Ethics Committee of Nihon Fukushi University. All methods were performed in accordance with the relevant guidelines and regulations. Participants were excluded if they: (1) had lower-extremity contracture; (2) had undergone surgery on their back or lower extremities; (3) had neurological disorders; (4) took hormones or muscle-affecting drugs; and/or (5) regularly engaged in competitive sports or resistance, aerobic, or flexibility training. The participants’ mean (± standard deviation) age, height, body mass, and body mass index were 21.7 ± 0.9 years, 170.7 ± 6.6 cm, 63.5 ± 11.2 kg, and 21.8 ± 3.3 kg/m^2^, respectively. Participants were asked to maintain their normal dietary habits and refrain from vigorous physical activity the day before and immediately before the experiment.

### Experimental design

**Figure 6:**
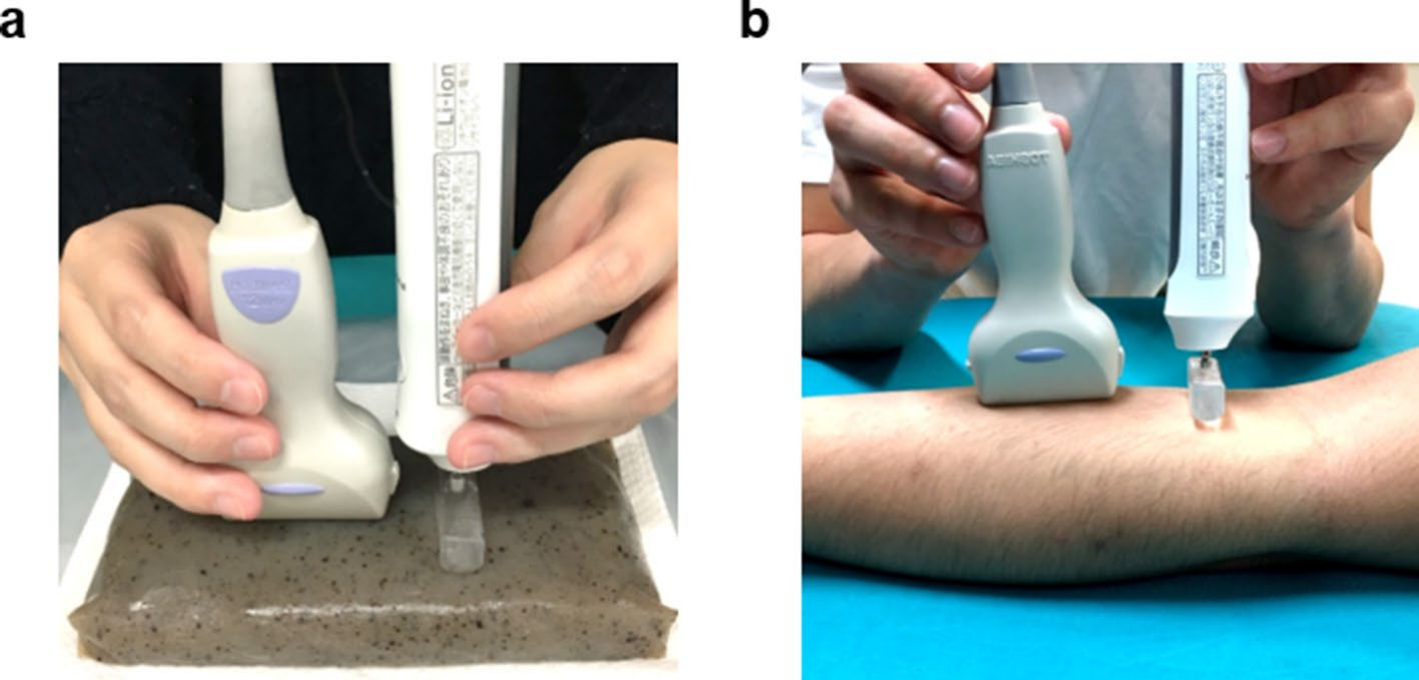
Photograph of the experiment. **(a)** Photograph of the phantom experiment. The phantom in the photograph is a commercially available jelly made from konjac yam routs. **(b)** Photograph of the in vivo experiment using forearm skeletal muscle.

Shear wave velocities were measured in phantom gels (konjac and agar) and skeletal muscles (right biceps brachii, flexor carpi radialis, semitendinosus, biceps femoris, medial gastrocnemius, and tibialis anterior) twice per day for 2 days, with a between-measurement interval of 1 day. During measurement of phantom gels, two investigators measured shear wave velocity. Phantom gels with different concentrations of agar (1%, 2%, 3%, 4%, and 8%) were mixed with sterilized milk (Table 2). The probe of an ultrasonic diagnostic device was placed directly over the measurement point and the excitation point of the vibrator was positioned next to the probe (Fig. 6). Before measurement, the probe position and excitation point were carefully adjusted, while the wave fronts of shear waves were monitored. In addition, the investigator arranged his posture to ensure maintenance of consistent application force to the vibrator and probe during each measurement. In most instances, the investigator’s elbows were placed on the bed to maintain a stable posture. This positioning was maintained and 3–5 measurements were performed. During measurements of skeletal muscle, the probe position and excitation point were parallel to the long axis of the muscle fibers; this positioning was adjusted to ensure that shear waves clearly propagated in the muscle fiber direction. The elasticity of the middle portion of the muscle was examined and adjusted with reference to the recommended placement of surface electrodes for standardized electromyography measurement^53,54^. The measurement position was determined based on a body surface landmark. If the positions of the probe and vibrator varied between measurements, the measurement location and shear wave propagation would have differed, resulting in inconsistent measurements. Therefore, the positions of the probe and vibrator were marked on the body surface; the probe and vibrator were positioned at the same site for each measurement. Furthermore, a B-mode image acquired during the first measurement was used as a reference image. Measurements were performed on B-mode images; characteristic points of the fascia, bone, nerves, and blood vessels were kept consistent as much as possible.

### Statistical analysis

Statistical analyses were performed using the Statistical Package for the Social Sciences version 15.0 (SPSS Inc., USA). Distribution normality was assessed using the Shapiro–Wilk test. CV values were calculated to indicate measurement reproducibility and reliability^55^. To investigate the relationship between agar gel concentration and shear wave velocity, Pearson’s correlation analysis was performed. Data were compared using a paired t-test. Differences between conditions were considered statistically significant when P values were < 0.05. ICC (1,1) and ICC (2,1) values with 95% confidence intervals were calculated to determine intra-day, day-to-day, and inter-operator reliabilities. Guidelines from Landis and Koch^56^ were used to interpret reliability values as follows: 0.0–0.2 indicated slight agreement; 0.21–0.40 indicated fair agreement; 0.41–0.60 indicated moderate agreement; 0.61–0.80 indicated substantial agreement; and 0.81–1.0 indicated near-perfect or perfect agreement.

## Supplementary Information

Supplementary Figure S1.

## Data Availability

All data needed to evaluate the conclusions in the paper are present in the paper, supplementary material, and/or references cited within.
