# Supplementary material for: Application of the novel estimation method by shear wave elastography using vibrator to human skeletal muscle
Source: Sci Rep. 2020 Dec 17;10:22248. doi: 10.1038/s41598-020-79215-z (PMC7747727; doi:10.1038/s41598-020-79215-z)
Supplement: Supplementary file 1 — Supplementary Figure S1. [file 41598_2020_79215_MOESM1_ESM.docx]

**Application of the novel estimation method by shear wave elastography using vibrator to human skeletal muscle**

**Authors:**

Wakako Tsuchida,^1,*^ Yoshiki Yamakoshi,^2,*^ Shingo Matsuo,^3^ Mayu Asakawa,^4^ Keita Sugahara,^5^ Taizan Fukaya,^6,7^ Eiji Yamanaka,^8^ Yuji Asai,^3^ Naotaka Nitta,^1^ Toshihiko Ooie,^1^ Shigeyuki Suzuki^9^

**Affiliations:**

^1^Health and Medical Research Institute, Department of Life Science and Biotechnology, National Institute of Advanced Industrial Science and Technology (AIST), 2217-14 Hayashi-cho, Takamatsu, Kagawa, 761-0395, Japan

^2^Graduate School of Science and Technology, Gunma University, 1-5-1, Tenjin-cho, Kiryu, Gunma 376-8515, Japan

^3^Department of Rehabilitation, Faculty of Health Sciences, Nihon Fukushi University, 26-2 Higashihaemi-cho, Handa, Aichi 475-0012, Japan

^4^Department of Rehabilitation, Matsunami General Hospital, 185-1 Dendai, Kasamatsu-cho, Hashima, Gifu, 501-6062, Japan

^5^Medical Corporation Sanaikai, 2-2-1 Tada, Obama, Fukui, 917-0026, Japan

^6^Institute for Human Movement and Medical Sciences, Niigata University of Health and Welfare, 1398 Shimami-cho, Kita-ku, Niigata 950-3198, Japan

^7^Department of Rehabilitation, Kyoto Kujo Hospital, 10 Karahashirajoumon-cho, Minami-ku, Kyoto 601-8453, Japan

^8^Department of Rehabilitation Medicine, Tokyo Bay Rehabilitation Hospital, 4-4-1 Yatsu, Narashino City, Chiba, Japan

^9^Department of Health and Sports Sciences, School of Health Sciences, Asahi University, 1851 Hozumi, Mizuho, Gifu 501-0296, Japan

*Correspondence to: yamakoshi@gunma-u.ac.jp, w-tsuchida@aist.go.jp


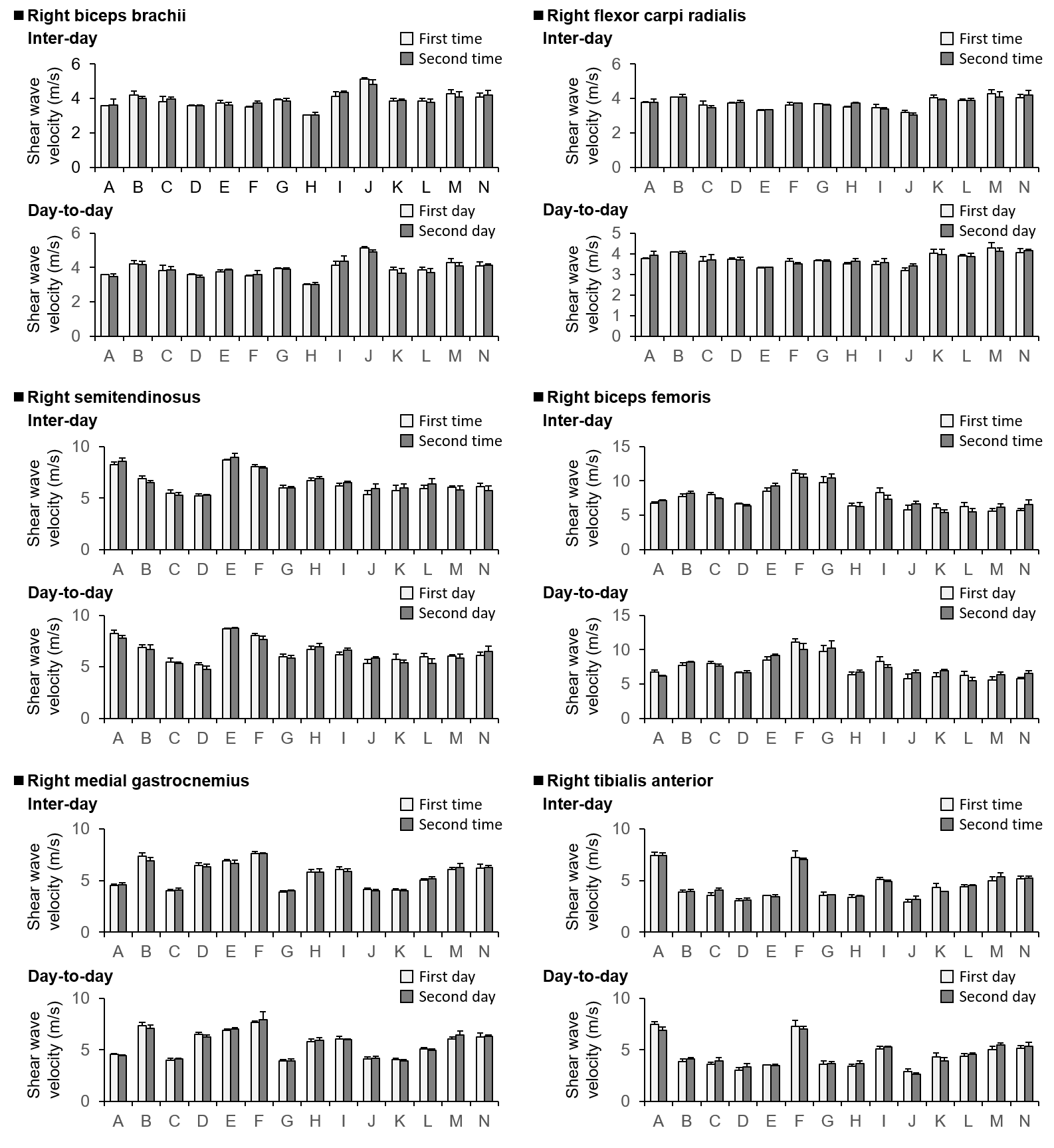


**Supplementary Figure 1.** Results of each participant’s skeletal muscle shear wave velocity. The figure shows mean ± SD of the skeletal muscle shear wave velocity of participants A–N. The right biceps brachii, flexor carpi radialis, semitendinosus, biceps femoris, medial gastrocnemius, and tibialis anterior were measured twice per day for two days with an interval of one day (three times per trial).
